# Association Between Sensation Seeking and Fear Response: Interventional Study of Personality and Behavior Using a Virtual Reality Heights Simulation

**DOI:** 10.2196/73785

**Published:** 2025-11-04

**Authors:** Rebecca C Daugherty, Christina S Jacksack, Becca J Daye, Brandon G Oberlin

**Affiliations:** 1 Department of Psychology, School of Science, Indiana University Indianapolis, 420 University Blvd, Indianapolis, IN, United States; 2 Department of Psychiatry, Indiana University School of Medicine, 355 W 16th Street, Indianapolis, IN, 46202, United States, 1 317-963-7215

**Keywords:** behavioral psychology, immersion, immersive virtual reality, novelty seeking, real-life responses, phobia, risk-taking, simulation

## Abstract

**Background:**

Immersive virtual reality (VR) technology presents digital simulations that create the sense of an actual experience. VR simulations are persuasive enough to elicit physiological reactions that mirror real-world responses. Prior research suggests that fear responses and sensation seeking are inversely correlated, but that work largely relies on self-reported outcomes and hypothetical scenarios.

**Objective:**

To more closely model real-world phenomena, we tested for inverse associations using an experiential height exposure simulation and a behavioral task for sensation seeking. We tested these associations comprehensively by using multiple methods for convergent evidence.

**Methods:**

A total of 57 healthy undergraduates participated in an interventional study that included an anxiety-inducing VR simulation, behavioral tasks, and personality inventories. The VR paradigm (Richie’s Plank) prompted users to walk across and step off a plank at the top of a skyscraper. This simulation of extreme height exposure and falling was intended to evoke fear. Physiological recordings and self-reported state anxiety were collected prior to and during the experience. Behavioral sensation seeking was quantified using an olfactory choice task offering a “boring” or “exciting” (risky) option varying in intensity and pleasantness. Evoked fear was calculated as the difference between the calm (pre-plank) and provoked fear state (standing on the plank), with correlations performed between evoked fear and personality and behavioral measures. The false discovery rate was set to *q*<.05, with analyses conducted in SPSS.

**Results:**

The VR experience evoked self-reported fear (*P*<.001) and physiological arousal (*P*<.006 for heart rate and *P*<.017 for respiration). Acrophobia correlated with self-reported fear in men and women (*P<*.01). Behavioral sensation seeking negatively correlated with both self-reported fear (*P*=.02) and increased heart rate in men (*P*=.02). Behavioral and self-reported sensation seeking were uncorrelated (*P*=.89). Self-reported fear was uncorrelated with physiological fear responses (*P*>.46).

**Conclusions:**

VR simulations can produce lifelike responses to scenarios that are impractical to test in reality. Our demonstration of an experiential manipulation negatively correlating with sensation seeking behavior in men increases confidence in other findings from studies using more traditional methods. Our use of VR along with objective measures, for example, behavioral tasks, and subjective measures, for example, self-report, confirm the effectiveness of these tools to investigate behavioral health topics. Our findings further suggest that sex is an important intervening factor for fear and sensation seeking and that additional study is warranted. This study highlights the potential of VR to expand convergent validity more broadly with other traits and paradigms. Finally, VR technology permits presenting highly abstract or improbable scenarios, thus expanding the range of topics for behavioral investigations. Given the ever-wider adoption of immersive therapeutics in the clinic, VR research will continue to facilitate the study of biobehavioral outcomes and interactions with personality factors and advance actionable knowledge for clinical applications.

## Introduction

Immersive virtual reality (VR) produces the functional and behavioral equivalence of actually being in a place, known as “presence” [1-5]. Immersion arises from high-fidelity stereoscopic visual and 3D audio stimuli that respond to user inputs; together, these create an “inclusive, extensive, surrounding, and vivid illusion of reality” [6]. Germane to clinical research, VR can deliver experiences that feel authentic but would otherwise be too dangerous, costly, or impractical to administer in a laboratory setting [1]. Until recently, research on evoked fear responses generally relied on affective pictures, video, and threat scenarios [7-9]. Extending prior research demonstrating that increasing immersive properties increased evoked responses [10,11], VR technology offers the promise of delivering experiences that are interactive, vivid, powerful, customizable, and standardized for studying clinically relevant outcomes.

Fear is an arousal response to threat stimuli, whereas anxiety occurs while approaching or anticipating a threat [12]; however, the distinction between fear and anxiety becomes blurred as the threat gets closer or more certain [13]. Unreasonable fear and anxiety elicited by particular stimuli can manifest as specific phobias, which occur in ~10% of the population and are highly comorbid, especially with anxiety, mood, and personality disorders [14]. The fear of heights is the second most common among these specific fears, with a prevalence of 4.5% [14], and is twice as common in women than in men [14,15]. The physiological fear response is mediated by the Fight/Flight/Freezing System and autonomic arousal, which can be readily quantified using altered heart rate [12,16]. Longitudinal evidence suggests that evoked physiological responses are more reliable than baseline physiological measures [17]. Further, physiological responses appeared to be more sensitive than self-reported valence to simulated threats when those threats were more lifelike and created by computer-generated graphics [11]. Immersive VR compared to 2D versions of provocative scenes evoked stronger physiological and emotional responses, coinciding with a stronger sense of presence [10,18]. Prior work indicates that virtual height simulations effectively elicit fear-related arousal [5,19]. We reasoned that a realistic experiential fear provocation would be highly appropriate for investigating associations with other objectively measured behavioral outcomes.

Sensation seeking, defined as “the seeking of varied, novel, complex, and intense sensations and experiences, and the willingness to take physical, social, legal, and financial risks for the sake of such experience” [20], is a putative stress buffer [21]. It is negatively associated with anxiety [19,22,23], risk perception [22,24], and fear responses [25]; however, the associations differ by sex (particularly on the Experience Seeking dimension) [26]. Unsurprisingly, fear-inducing activities (eg, hang-gliding, BASE jumping) attract high sensation seekers [27,28]. Moreover, many “extreme sports” (eg, bungee jumping, skydiving, ultralight piloting) evoke the fear of falling, a powerful and common “natural fear” in humans [29].

Sensation seeking is traditionally measured with self-report inventories [30,31]. To quantify sensation seeking and overcome some of self-report’s intrinsic limitations, such as social desirability, self-awareness, cultural limitations, and criterion contamination [32-35], we created a behavioral task that models the key aspects of this trait [36]. The Aroma Choice Task (ACT) quantifies sensation seeking behavior as a binary choice between olfactory stimuli varying in intensity, novelty, and riskiness using actual sensory experiences presented in real time. Sensation-seeking behavior in this task correlates with self-reported sensation seeking [36], reward-related brain activation [37], and alcohol-induced shifts in reward preference [38]. Given the body of work suggesting that studies relying on low-realism tasks, hypothetical choices, and self-report questionnaires limit neurobehavioral [39] and behavioral genetic [40] conclusions, our objective sensation seeking task quantifies the behavioral trait as described by Zuckerman [30].

While virtual height simulations consistently elicit physiological reactions [5,19,41,42], only a few studies have examined relationships between behavior and sensation seeking; however, in VR [19,43], no studies, to the best of our knowledge, have tested for associations of physiological fear responses and behavioral sensation seeking. We expect more ecologically valid manipulations (ie, greater resemblance to real-world experience) of fear and anxiety, such as a realistic VR simulation of extreme heights, paired with objective behavioral measures of sensation seeking to reveal more generalizable findings for clinical research. Here, we elicit fear responses with an immersive interactive height exposure simulation [44] and test for associations with sensation seeking behavior. We quantify fear responses as subjective (evoked state anxiety) and objective (evoked physiological arousal). We hypothesize that (1) the virtual height simulation will increase evoked state anxiety and physiological arousal, (2) evoked state anxiety and physiological fear responses will be positively associated, (3) height anxiety will correlate with evoked state anxiety, (4) behavioral sensation seeking and self-reported evoked state anxiety will be negatively associated, and (5) behavioral sensation seeking and physiological fear response will be negatively associated. We also explore potential correlations between behavioral and self-reported sensation seeking.

## Methods

### Ethical Considerations

The Indiana University Institutional Review Board approved all recruiting and study procedures (protocol number 17398). Students provided informed consent before study participation. Privacy and confidentiality were maintained for all participants, and compensation was awarded in the form of class credit.

### Participants

A total of 57 healthy undergraduate students were recruited from an urban midwestern university via online listings through the university’s Sona Systems human subjects pool; course credit was provided for participation. Students provided informed consent before study participation. Exclusions included poor sense of smell, extreme sensitivity to odors or volatile chemicals, chronic or current asthma, pregnancy or nursing, or the use of a nasally administered medication (excepting steroids). Physiological data collection devices were unavailable in the beginning of the study, so those data were not collected from 21 participants. Technical challenges additionally limited data collection for heart rate and respiration (n=1), heart rate only (n=2), and respiration only (n=1). At least 1 participant did not feel well enough to complete the study and provided only demographic and personality data.

### Procedure

**Figure 1.**
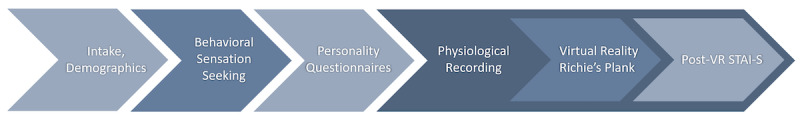
Experimental procedures. Undergraduate student participants were tested in the same manner in a pre-post intervention design. Intake included screening and informed consent, followed by a demographics questionnaire (eg, age, sex, childhood income, race). All procedures were completed within 60 minutes and were conducted in the same experiment room (pictured in Figure 2A and B). Behavioral sensation seeking was assessed with the Aroma Choice Task (ACT). Personality questionnaires included the State-Trait Anxiety Inventory for States (STAI-S), Acrophobia Questionnaire-Anxiety, and Zuckerman Kuhlman Personality Questionnaire (ZKPQ). The Richie’s Plank Experience is described in detail in the text. Physiological data (heart rate and respiration) were recorded throughout virtual reality (VR).

Prior to the VR experience, participants provided demographic information and self-reports of anxiety, height-specific anxiety (major component of acrophobia), and impulsive sensation seeking. All self-report inventories were administered via Qualtrics. Participants also completed the behavioral sensation-seeking task. Participants were then guided through an immersive fear-inducing VR simulation of height exposure, with physiological recording initiated before the VR experience and continuing until the VR task was completed. Self-reported state anxiety was measured again near the end of the fear induction while in VR. Figure 1 illustrates the experimental procedures in a timeline format.

**Figure 2.**
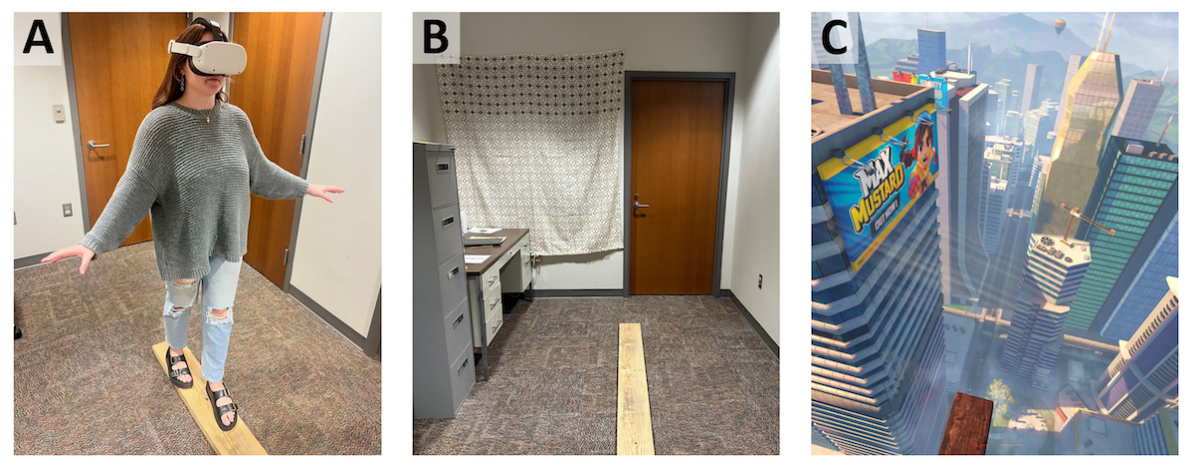
Experimental environment and display. (**A**) All experimental procedures were conducted in the private quiet room shown, with participants’ initial steps corresponding to the tactile experience of walking on a plank. (**B**) The researchers’ view of the room and plank, and (**C**) the visual effects as displayed to participants. Wind noise corresponding to high altitudes was played through the headset speakers while walking on the plank. The tactile, visual, and auditory elements integrated for an immersive visceral simulation of walking on a plank from a city building at extreme height. Participants were oriented facing the plank at the onset of the paradigm.

### Self-Report Inventories

#### Anxiety

Changes in self-reported anxiety before and during the experience indexed evoked fear. The State-Trait Anxiety Inventory for states (STAI-S) is a 20-item questionnaire rated on a Likert-type scale anchored by “not at all” (1) to “very much so” (4). Participants report how they currently feel (eg, *“I feel frightened”*). Total scores range from 20 to 80, with 80 indicating the highest anxiety levels [45]. This questionnaire assessed baseline and evoked anxiety, with the subtracted (Pre-Plank vs Plank) difference quantifying evoked anxiety. We changed the administration of the STAI-S during data collection to better capture the emotional state while still in VR. The first 21 participants completed the inventory on a laptop following the experience, but the last 36 participants were queried verbally while still in the headset (standing on the end of the virtual plank), and responses were recorded by the experimenter. “Plank,” the post condition, refers to both ways of measurement. This change was provoked by the concern based on behavioral observation that STAI-S responses following the experience might be substantially influenced by relief, that is, anxiety alleviation, upon the termination of the fear-inducing experience. To ensure consistency, both the Pre-Plank and Plank STAI-S inventories were conducted verbally following this change.

#### Height-Specific Anxiety

The Acrophobia Questionnaire-Anxiety is a 20-item questionnaire that poses hypothetical height-related fear scenarios (eg, “*looking down a stairway from several flights up*”) and collects responses on a 7-point Likert-type scale anchored by “not at all anxious; calm and relaxed” (0) to “extremely anxious” (6). Total scores ranged from 0 to 120 with higher scores indicating greater levels of anxiety specifically related to heights, or acrophobia [46].

#### Impulsivity and Sensation Seeking

The Zuckerman Kuhlman Personality Questionnaire is a 50-item forced-choice inventory posing self-descriptive statements (eg, “*I often do things on impulse*”). The 5 subscales are impulsive sensation seeking, neuroticism-anxiety, aggression-hostility, activity, and sociability. Possible scores on each ranged from 1 to 10, with 10 indicating a high presence of the trait [47].

### Behavioral Sensation Seeking

The Aroma Choice Task (ACT) is a validated behavioral test of sensation seeking that measures the relative preference for an intense, novel, varied, risky option versus a mild, safe, “boring” option, with odorants delivered in real time. Participants are instructed:

*For the next 12 minutes, you will make choices about some smells. The choice labeled ‘Standard’ will likely be mild and pleasant. The choice labeled ‘Varied’ will likely be stronger and pleasant, but there is a chance that it will be unpleasant. Upon making a choice, please inhale deeply through your nose to receive the aroma.*

Choice ratio, the percentage of “Varied” choices out of a total of 20 binary choice trials (range: 0%‐100%), yields a single behavioral index reflecting behavioral sensation seeking (designed after self-reported sensation-seeking trait descriptions) [30,31]. The original ACT was developed with an air dilution olfactometer [36], but a simpler, manual version yielded analogous results [38]. We further modified the task to deliver 20 trials instead of the original 40, as our prior work indicated that the first 20 trials accurately capture the trait with lower participant burden [36].

### Physiological Recording

Heart rate, respiration, and skin conductance were collected for 36 participants using the BioRadio (Great Lakes Neurotechnologies; Cleveland, OH, USA) and skin electrodes plus a respiratory inductance plethysmography belt, with data logged on a laptop computer. At least 1 researcher closely assisted the participant during VR to prevent collisions and stumbles. A second researcher entered live event markers (single keystrokes) that were logged in the BioRadio data stream as the participant progressed through the phases of the VR experience. Standardized breathing exercises (eg, Balban et al [48]) prior to VR were intended to mitigate physiological variability between participants. The “Pre-Plank” measurements included the anticipatory elevator ride up to altitude and the 20 seconds before stepping onto the plank (mean 0.74 [0.24] minutes; max 1.70). The “Plank” measurements comprised stepping onto the plank after the 20 seconds was complete and then walking the length of the plank and off into freefall (mean 1.88 [0.60] minutes; max 2.85). Skin conductance data were not analyzed.

### Virtual Reality

The fear of falling from heights is an “innate” fear, that is, nonreliant on associative conditioning [29] or locomotion experience [49], and nearly universal [50], meaning it is highly generalizable and reliable for eliciting potent fear responses. An immersive height exposure simulation was delivered on the Meta Quest 2 head-mounted VR display. The Meta Quest 2 features 6 degrees of freedom, high refresh rate (up to 120 Hz), and adjustment for interpupillary distance [51,52]. These features, combined with limited movement and short VR duration (mean 2.90 [0.58] minutes, max 3.90), minimized the risk of VR sickness [53]. Nonetheless, participants were informed about the risk of VR sickness and were encouraged to notify the researcher at any time to end the simulation. No participants complained of VR sickness or feeling unwell due to the VR experience.

The immersive height exposure simulation was the “Richie’s Plank Experience” [44] delivered with a Meta Quest 2 head-mounted VR display. This simulation is widely used in VR research on fear responses and behavior [43,54,55]. It has also been used to study risk-taking [56] and suicide willingness [57] and is even proposed as a psychological preparation for psychedelic experience [58]. The paradigm simulates an elevator ride to the top of a tall building, and upon the door opening, presents a cityscape view from heights (~80 stories). Participants walk onto, down the length of, and off the end of a wooden plank protruding from the skyscraper. The height illusion is conveyed by audio and visual simulation of extreme exposure to open space and reified with wind noise and birds flying below the participant. Immersion was maximized with haptic feedback from a real wooden plank (6’ long “2×8” [2 m×4 cm×19 cm]) spatially registered to the virtual plank. The wooden plank was slightly warped and thus creaked and shifted with human weight. Although verbal instructions were provided as needed, researchers refrained from unnecessary verbal or physical interaction during the experience to preserve presence. A simulated participant (the first author) walks on the plank (Figure 2A) in the center of partitioned office space (Figure 2B), with the participants’ view at heights illustrated in Figure 2C.

### Analytical Strategy

Physiological data collected by the BioCapture (Great Lakes Neurotechnologies; Cleveland, OH, USA) software were modeled in VivoSense (version 3.4). VivoSense calculated the means of heart rate and respiration rates in 10-second bins. Changes in physiological measures were calculated as the average rate during anticipation of stepping on the plank (Plank) minus the baseline average (Pre-Plank). All statistical tests were performed in SPSS v29.0.2.0 (IBM). Analyses were stratified by sex as prior work demonstrated important interactions by sex between anxiety and sensation seeking [26] and by sex and elements of sensation seeking [30,59]. VR-induced increases in anxiety and physiological arousal were tested with paired *t* tests (2-tailed) between baseline “Pre-Plank” and the moment of stepping off the plank “Plank.” Change scores were calculated for self-reported state anxiety and physiological measures (Plank minus Pre-Plank scores). These were tested for correlations with behavioral sensation seeking, each other, and height-specific anxiety. The potential effect of changing the STAI-S assessment method (computer vs verbal inside VR) was tested with independent-samples *t* tests (2-tailed). We tested for differences in participant characteristics of those with physiological recording versus without to assess potential effects on interpretation. *t* tests (2-tailed) of inhomogeneous variance (Levene test) were performed using the Welch *t* test (2-tailed). The false discovery rate was controlled with the Benjamini-Hochberg procedure [60], and limited to 5% (*q*<.05), by setting *α*=.0278 for individual tests in a priori hypotheses.

## Results

### Demographics and Personality

Tests evaluating baseline sex differences (*χ*^2^ for nominal, *t* test [2-tailed] for continuous) reported were uncorrected and descriptive in nature. All in-text data are reported as mean (SD). Women reported higher baseline levels of anxiety than men did (neuroticism-anxiety, state anxiety, and height-related anxiety, *P*<.03; Table 1). No other sex differences were detected.

**Table 1.** Participant characteristics: demographics and personality (n=57).

Characteristic	Women (n=40)	Men (n=17)
Age (y), mean (SD)	20.23 (2.94)	20.06 (2.19)
Childhood income^a^ (US $), median (IQR)	86,000 (35,000-127,000)	86k (35,000-141,000)
Race, n (%)
American Indian	1 (3)	0 (0)
Asian	6 (15)	1 (6)
Black	6 (15)	2 (12)
Other/Unknown^b^	6 (15)	2 (12)
White	21 (53)	12 (71)
ZKPQ^c^, mean (SD)
Impulsive sensation seeking	3.82 (2.41)	4.35 (2.57)
Neuroticism-anxiety	5.50 (3.11)^d^	2.06 (2.08)
Aggression-hostility	4.24 (1.84)	3.88 (2.20)
Activity	5.05 (2.68)	4.41 (3.24)
Sociability	4.05 (2.52)	3.65 (3.18)
STAI-S^e^, mean (SD)	34.26 (9.31)^f^	28.59 (7.87)
Acrophobia questionnaire-anxiety, mean (SD)	55.60 (18.78)^g^	43.12 (19.69)

a Reported as median and interquartile ranges representing the geometric means of the inventory ranges (<US $10,000, $10,000-$24,000, $25,000-$49,000, $50,000-$74,000, $75,000-$99,000, $100,000-$199,000, $200,000-$500,000, >$500,000).

b Hispanic ethnicity: n=5 identified as Other/Unknown, n=3 as White, n=1 as American Indian. Hispanic ethnicity did not differ by sex or race (*χ*2, *P*>.71)

c ZKPQ: Zuckerman Kuhlman Personality Questionnaire subscales.

d *t*_44.7_=4.83, *P*<.001.

e STAI-S: State Trait Anxiety, Inventory for states.

f *t*_54_=2.19, *P*=.03.

g *t*_55_=2.26, *P*=.03.

### Evoked Fear: Increased Anxiety

**Figure 3.**
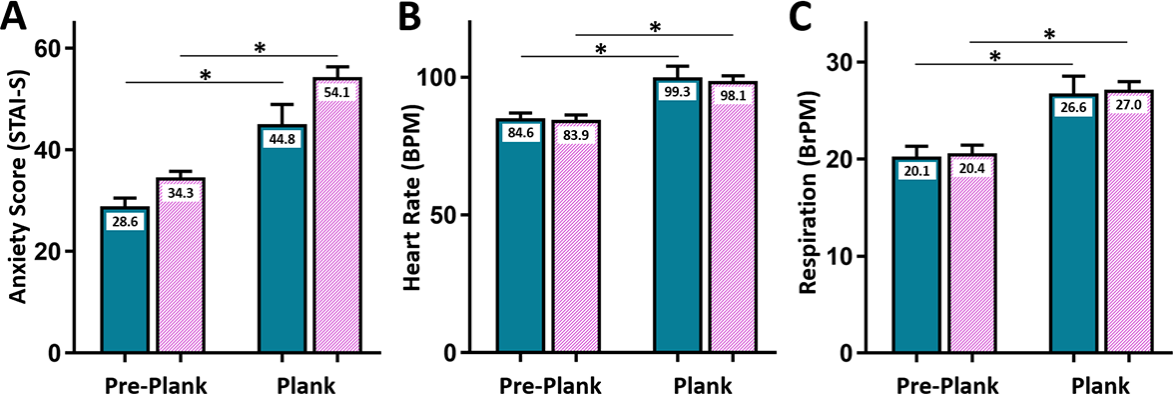
Effects of VR. Participants (solid cyan bars show the values for men, white/violet diagonal hash bars show the values for women) showed increased (**A**) anxiety states, (**B**) heart rate, and (**C**) respiration when on the plank relative to the Pre-Plank baseline. STAI-S=State trait anxiety inventory, State; BPM=beats per minute; BrPM=breaths per minute; *q<.05.

The VR experience increased anxiety in both men (*t*_16_=5.29, *P*<.001, 28.59 [7.87], and 44.76 [17.27], *q*<.05, Pre-Plank and Plank, respectively) and women (*t*_38_=9.50, *P*<.001, *q*<.05, 34.26 [9.31] and 54.05 [14.51]; Figure 3A). The increase in anxiety was not significantly different before versus after the data collection method change (for more details, see the Anxiety section; *P*=.75).

### Physiological Arousal

In the 36 participants from whom physiological data were collected, the VR experience increased heart rate in both sexes (*t*_8_=3.84, *P*=.005 for men *t*_23_=6.99, *P*<.001 for women; *q*<.05; Figure 3B). Similarly, the VR experience increased respiration in both sexes (*t*_8_=3.06, *P*=.02 for men and *t*_24_=5.35, *P*<.001 for women; *q*<.05; Figure 3C). Of note, characteristics (Table 1) did not differ between participants with physiological data collected versus those without, *q*>.05 (11 tests); *P*=.53, *P*=.046, *P*=.24, *P*=.51, *P*=.84, *P*=.88, *P*=.62, *P*=.60, *P*=.25, *P*=.50, and *P*=.61, corresponding to childhood income, age, impulsive sensation seeking, aggression-hostility, activity, sociability, neuroticism-anxiety, AQ-anxiety, STAI, sex, and race, respectively.

### Evoked Fear and Physiological Response

Fear (evoked state anxiety) did not correlate with changes in heart rate (*P*=.60 and .79 for men and women, respectively) nor respiration (*P*=.47 and .48) in the participants from whom physiological data were collected. Baseline anxiety was also uncorrelated with changes in heart rate (*P*=.70 and .29) and changes in respiration (*P*=.71 and .17).

### Acrophobia and Evoked Fear

**Figure 4.**
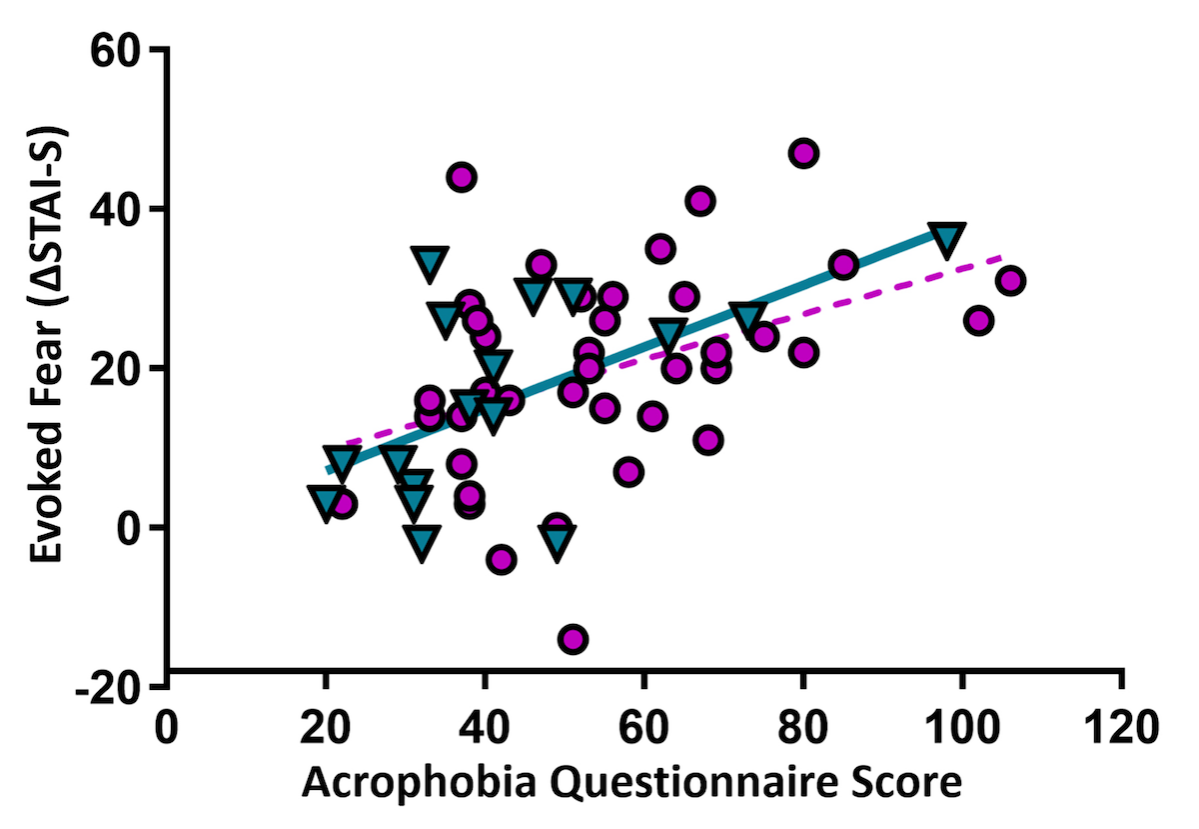
Height-related fear and fear response. Higher scores on the Acrophobia Questionnaire in both men (cyan triangles, solid line) and women (violet circles, dotted line) predicted greater anxiety reactivity to the virtual reality (VR) experience (*r*>.40, *P*<.011, *q*<.05). Regression lines illustrate strength and direction of associations. ΔSTAI-S: Plank minus Pre-Plank STAI-S scores.

Fear of heights (acrophobia) was correlated with evoked state anxiety in men (*r*(15)=.606, *P*=.01) and women (*r*(38)=.410, *P*=.009, *q*<.05; Figure 4).

### Behavioral Sensation Seeking and Evoked Fear

**Figure 5.**
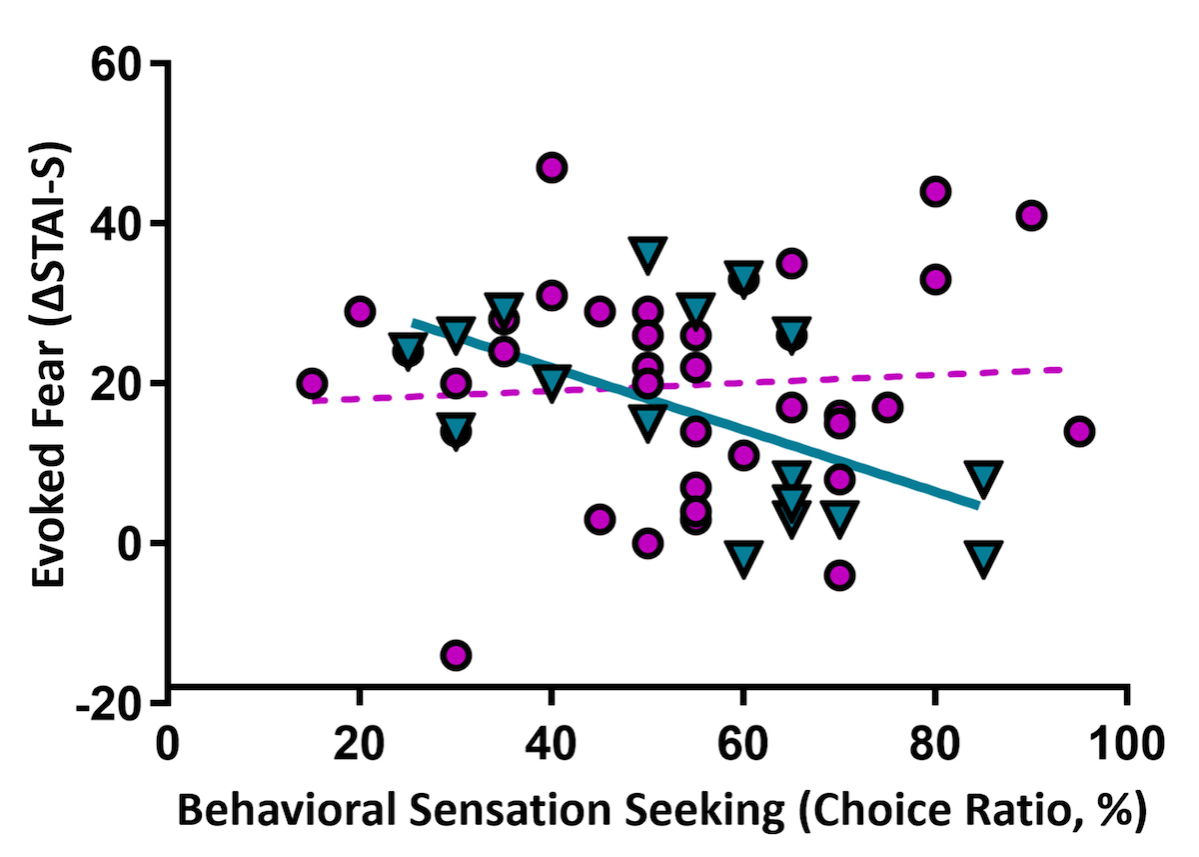
Sensation seeking and fear induction. Higher sensation seeking in men (cyan triangles, solid line) predicted lower anxiety response in virtual reality (VR*; r*=−.559, *P*=.02, *q*<.05), but not in women (violet circles, dotted line), *P*=.67. Regression lines illustrate strength and direction of associations. Choice ratio is the percentage of “varied” choices selected during the aroma choice task. ΔSTAI-S: Plank minus Pre-Plank STAI-S scores.

High intensity preference (ACT scores) negatively correlated with evoked state anxiety (Δ STAI-S) in men (*r*(15)=−.559, *P*=.02, *q*<.05), but not in women (*P*=.67; Figure 5). Preference for high intensity was not correlated with baseline STAI-S scores in women (*P*=.61), although there was a trend in men (*r*(15)=−.465, *P*=.06).

### Behavioral Sensation Seeking and Physiological Fear Response

**Figure 6.**
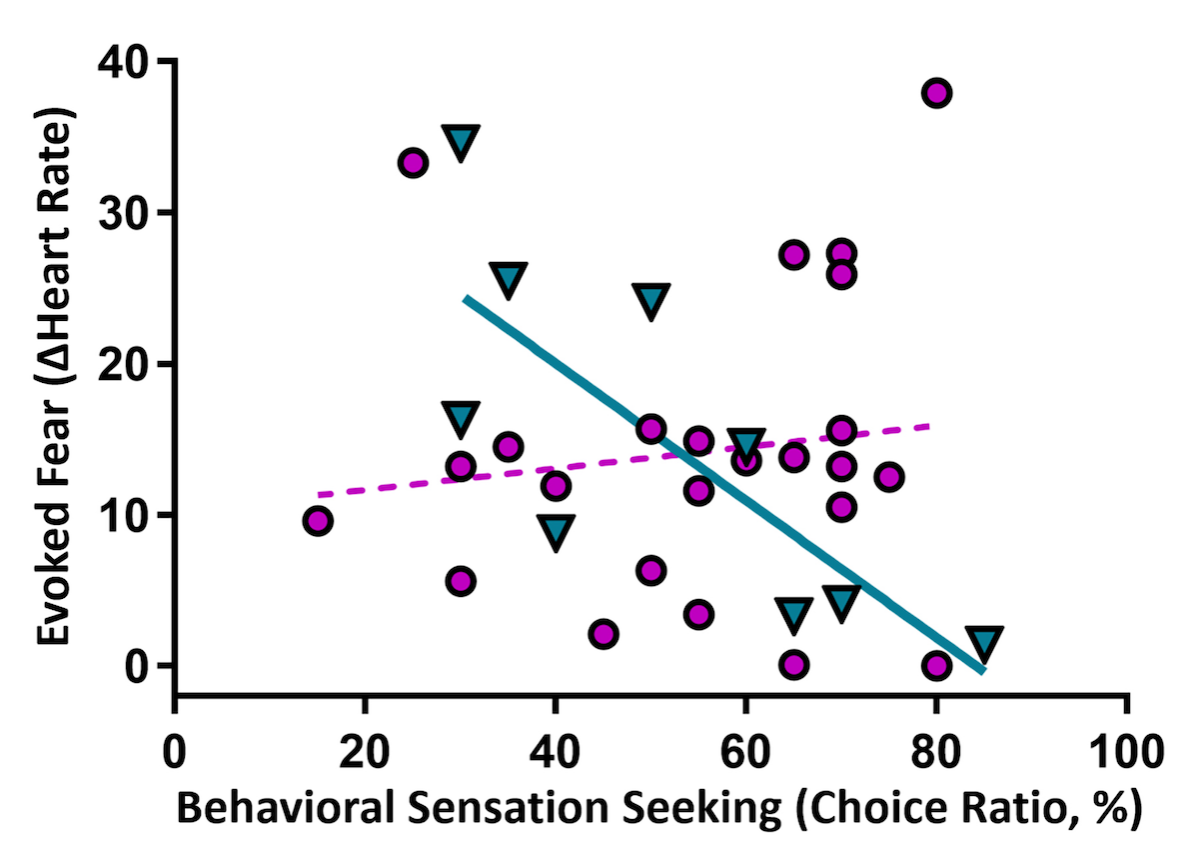
Sensation seeking and physiological fear response. Higher sensation seeking in men (cyan triangles, solid line) predicted lower heart rate reactivity to the virtual reality (VR) experience (*r*=−.771, *P*=.02, *q*<.05), but not in women (violet circles, dotted line), *P*=.54. Regression lines illustrate strength and direction of associations. Choice ratio is the percentage of “Varied” choices selected during the aroma choice task. ΔHeart Rate: Plank minus Pre-Plank heart rate measurements.

High intensity preference (ACT scores) negatively correlated with increased heart rate in men (*r*(7)=−.771, *P*=.02, *q*<.05), but not women (*P*=.54; Figure 6). Increased respiration was uncorrelated in both sexes (*P*>.28).

### Behavioral and Self-Reported Sensation Seeking

High intensity preference did not correlate with self-reported sensation seeking in men (*P*=.47) or women (*P*=.72); collapsing across sex did not permit detection of an association (*P*=.89). See Table 2 for correlations of personality and behavioral metrics (uncorrected).

**Table 2.** Systematic matrix of correlations.

1	2	3	4	5	6	7	8	9	10
ZKPQ^a^
1. ImpSS	—	0.082	0.286^b^	0.324^b^	−0.036	0.019	−0.173	−0.224	−0.027	0.212
2. Agg-Host	—	0.104	0.163	0.197	0.106	0.087	−0.134	0.238	0.349^b^
3. Act	—	0.202	0.210	−0.130	0.187	0.159	0.354^b^	0.127
4. Sy	—	−0.001	0.073	−0.097	0.059	0.229	0.261
5. N-Anx	—	−0.140	−0.332^b^	0.309^b^	−0.073	−0.142
Beh. SS (ACT^c^)										
6. Choice ratio	—	−0.111	−0.236	−0.012	−0.029
Reported anxiety
7. ΔSTAI-S	—	0.483^d^	−0.039	−0.034
8. AQ-Anx	—	−0.318	−0.128
VR physiology
9. HR Δ	—	0.137
10. Resp Δ	—

a ZKPQ: Zuckerman Kuhlman Personality Questionnaire subscales.

b Correlation is significant at *P*<.05.

c ACT: Aroma Choice Task.

d Correlation is significant at *P*<.01.

## Discussion

### Primary Findings

We found support for our hypotheses concerning VR’s capacity to evoke fear (self-reported changes in state anxiety and physiological arousal) and the association between evoked fear and fear of heights. We found support for the negative association between evoked fear and behavioral sensation seeking in men, but not women. This unexpected finding is potentially due to higher trait anxiety scores in women, that is, imposing a ceiling effect and making associations between elicited anxiety and other traits difficult to detect. Interestingly, evoked fear measures were uncorrelated. Behavioral and self-reported sensation seeking were uncorrelated. These findings suggest that VR experiences possess sufficient ecological validity to elicit subjective and objective fear responses mirroring responses to real-world scenarios. Moreover, responses to the digital experience reflect associations with other traits (acrophobia and sensation seeking).

This study demonstrates the potential of VR for neuroscience and clinical research. VR continues to offer considerable promise for clinical and research applications alike. The expanding reach of VR is facilitated by better immersion technology, decreasing cost, creative applications, and wider adoption. While VR has long been used to administer exposure therapy [61-63] and treat pain [64-66], emergent applications target increasingly abstract constructs [67,68]. Germane to this study, VR applications effectively treat various anxiety disorders (eg, specific phobias, social anxiety disorder, panic disorder) in randomized controlled trials, producing effects comparable to conventional treatment and significantly better than passive controls [69]. In addition to promising efficacy data, providers and patients appear eager to adopt VR methods, as suggested by 2 recent reports on integrating VR in clinical practice [70,71]. The immersive nature of this nascent technology permits new avenues of investigation and permits research on humans that would be dangerous or impractical to study with real stimuli. We believe that the potential of VR to unite human and animal paradigms heralds a new translational era wherein strictly controlled animal neuroscience experiments can be accurately replicated in humans. For example, the elevated plus-maze—the gold standard in rodent anxiety research—can be instantiated for human participants [19] to connect basic neuroscience in rodents with human behavioral data. Other behavioral paradigms widely used in rodents, such as conditioned place preference, can now be accurately reproduced in humans using VR [72], producing convergent findings in both research contexts. Thus, the role and relevance of VR for both laboratory research and clinical practice are expected to grow substantially in the near future. The use of VR permits testing extant theoretical knowledge in more lifelike settings and experiences, and when paired with behavioral tests, yields increasingly objective outcomes.

VR can add knowledge to mature bodies of research, such as approach-avoidance, by presenting realistic simulations in humans. Approach-avoidance describes behavior that orients organisms toward positive and away from negative stimuli, respectively [73]. Approach-avoidance tendencies are likely rooted in evolutionary factors, primarily through sexually divergent selection pressure [74]; that is, reproductive fitness optimized by exploratory behaviors in men [75,76] and harm avoidance in women [77,78]. The extremes of the approach-avoidance continuum are marked by exaggerated attention on reward or threat cues [79]. These tendencies emerge from overactive brain reward or motivational systems [80] and underregulated brain threat systems [81] for approach, and conversely, overactive brain threat systems [82] and underactive brain reward systems [83] for avoidance. Sensation seeking and fear represent aspects of approach and avoidance, that is, opposing processes that modulate threat responses, such that high sensation seekers are less physiologically responsive to threat stimuli than low sensation seekers. One study testing fear responses found that high sensation seekers showed no response to threatening stimuli (versus control stimuli), whereas low sensation seekers produced an 8-fold increase in electromyographic response to threats [25]. No group difference in self-reported emotional reactivity was detected, further supporting the value of objective measurements.

Men are higher sensation seekers than women (particularly thrill or adventure seeking and disinhibition) [84,85], but the relationship between sensation seeking and fear appears to differ by sex. Investigating this relationship as an interaction with sex, Blankstein [26] found that the Sensation Seeking Scale (SSS) [23] total score negatively correlated with anxiety reactivity (Activity Preference Questionnaire) total and subscales (Social and Physical) at *r*>.43, *P*<.01 in men, but not in women (*r*<.07). However, a similar study found a number of negative correlations between the Sensation Seeking Scale and anxiety-related items (S-R Inventory) in both sexes [86], indicating mixed results in detecting sex interactions with approach-avoidance correlations. The lack of consilience in prior work might be explained by either (1) dependence on self-report inventories, with self-reported fear and sensation seeking often incongruent with objective measures [38,87], or (2) high anxiety and low sensation seeking in women producing restricted ranges (ceiling and floor effects), making correlations difficult to detect. Both factors potentially contribute together to the divergence. Future well-powered studies, ideally using precise behavioral tasks, should clarify these possible associations.

We did not detect correlations between self-reported fear and physiological responses to height exposure. While this is perhaps a surprising result, prior work suggests that self-reported fear does not necessarily reflect biological responses. In a real-world test, participants’ self-reported fear of crime did not differ between walking down a dimly-lit path (vs well-lit control), but the dimly-lit path participants’ heart rate increased by 17% (*P*=.002), with that in the controls remaining unchanged [87]. Even patients with anxiety disorders do not accurately report the degree of physiological responses to stress in laboratory tests [88,89]. This lack of concordance may be explained by individual differences in interoceptive ability [90]. The disconnection between self-reported traits and objective measures is also found in behavioral assessments of impulsivity [91], empathy [92], and risk preference [93], suggesting that the incongruence extends well beyond fear and anxiety. A recent report on associations between interoceptive ability and autobiographical memory [94] indicates that interoceptive perception (physical self-awareness) relates to episodic recall (cognitive self-awareness) and suggests the intriguing possibility that individual differences in these domains may be governed by some larger self-awareness meta factor.

Behavioral seeking and self-reported sensation seeking were uncorrelated in this sample. Existing findings offer scant data on this association due to the absence of behavioral sensation seeking tasks. Related traits such as impulsivity and risk-taking reveal low agreement between behavioral and self-reported assessment; for example, various measures of impulsivity are correlated at *r*=~0.1 in meta-analysis [91], and practically no relationship is observed between risk-taking measures [95]. Low reliability is observed in behavioral task data across domains [96]; in parallel, self-reported data suffer from various serious forms of bias [97]. The “jingle fallacy” (conflating interpretation of 2 measures because they have the same name) [98] exacerbates this problem. While the lack of convergence between behavioral and self-reported findings is perhaps unintuitive, this divergence between self-reported and behavioral data represents opportunities to discover additional features of personality traits. That is, important features of a given trait may not be fully captured by any single task or inventory.

### Limitations

Some limitations should be acknowledged. First, the sample would benefit from more power. The homogeneity of the sample—reflecting typical undergraduates—is predominately female and White, precluding well-powered direct comparisons by sex and potentially limiting generalizability. Generalizability would be enhanced by a community sample with a more even sex distribution and a larger range of age and socioeconomic status. Finally, the truncated sample of subjects providing physiological data was suboptimal, as physiological data were only collected from 36 of the 57 participants, although the absence of significant differences between the subsamples somewhat mitigates this concern.

### Conclusions

The current report demonstrates the potential use of VR for neuroscience and clinical research. Beyond research and education, VR is now well established as a clinically valuable tool in health care. The combination of using VR, objective measures (physiological recordings and behavioral tasks), and subjective measures (self-report) to investigate a behavioral health topic allows more rigorous investigation than any one of these approaches alone. Through these measures, we confirmed associations between self-reported experience and physiological fear in response to heights, in addition to behavioral patterns and personality related to sensation seeking. Further evidence of the disconnection between objective and self-reported methods was found, although this was perhaps unsurprising. We expect ever-wider adoption of VR applications and objective measures in the clinic and continued expansion in the laboratory research domain.

## Abbreviations

ACT: Aroma Choice Task
STAI-S: State Trait Anxiety Inventory-State
VR: virtual reality
